# Structural and biological features of a novel plant defensin from *Brugmansia* x *candida*

**DOI:** 10.1371/journal.pone.0201668

**Published:** 2018-08-02

**Authors:** Siriporn Kaewklom, Mathira Wongchai, Sawang Petvises, Warunee Hanpithakphong, Ratchaneewan Aunpad

**Affiliations:** 1 Graduate Program in Biomedical Sciences, Faculty of Allied Health Sciences, Thammasat University, Pathum Thani, Thailand; 2 Department of Medical Technology, Faculty of Allied Health Sciences, Thammasat University, Pathum Thani, Thailand; 3 Mahidol-Oxford Tropical Medicine Research Unit, Faculty of Tropical Medicine, Mahidol University, Bangkok, Thailand; Seconda Universita degli Studi di Napoli, ITALY

## Abstract

Data from both the laboratory and clinic in the last decade indicate that antimicrobial peptides (AMPs) are widely regarded as potential sources of future antibiotics owing to their broad-spectrum activities, rapid killing, potentially low-resistance rate and multidirectional mechanisms of action compared to conventional antibiotics. Defensins, a prominent family of AMPs, have been found in a wide range of organisms including plants. Thailand is a rich source of plants including medicinal plants used therapeutically, however there is no report of defensin from among these plants. In this study, a novel plant defensin gene, *BcDef*, was successfully cloned from *Brugmansia* x *candida* (Bc). *BcDef* cDNA was 237 bp in length, encoding 78 amino acids with a putative 31-amino acid residue signal peptide at the N-terminal followed by the mature sequence. BcDef shared high sequence identity (78–85%) with Solanaceae defensins and belonged to the class I plant defensins. From homology modeling, BcDef shared a conserved triple stranded β-sheet (β1-β3) and one α-helix (α1) connected by a loop (L1-L3). BcDef1 peptide, designed from the γ-core motifs of BcDef located in loop 3, showed antibacterial activity against both Gram-positive and Gram-negative pathogens with the lowest MIC (15.70 μM) against *Staphylococcus epidermidis*. This peptide affected cell membrane potential and permeability, and caused cell membrane disruption. Moreover, BcDef1 also exhibited antioxidant activity and showed low cytotoxicity against mouse fibroblast L929 cells. These findings may provide an opportunity for developing a promising antibacterial agent for medical application in the future.

## Introduction

Antimicrobial peptides (AMPs) are small (15–50 amino acid) peptides that contribute to host defense within the innate immune system in numerous organisms and have broad-spectrum antimicrobial activity [1]. AMPs have shown antagonistic activity against Gram-positive and Gram-negative bacteria, fungi, viruses and parasites [2]. Moreover, they showed antibacterial activity against antimicrobial drug-resistant bacteria such as penicillin-resistant *Escherichia coli* [3], multidrug resistant *Pseudomonas aeruginosa* [4], *Klebsiella pneumoniae* and *Acinetobacter baumannii* [5]. A tremendous effort has been devoted to the discovery and use of antimicrobial peptides from natural sources including plants. After exposure to a diverse array of pathogens, plants produce AMPs which can inhibit the growth of microbial pathogens as part of their self-defense mechanisms. Interestingly, most AMPs rapidly kill bacterial cells through their actions on the entire cytoplasmic membrane such as magainin 2, cecropin P1 or PR-39 which can kill bacteria in 15–90 minutes [6]. They also show a broad range of effectiveness. Therefore, it is believed that the development of AMP-resistance by bacteria is very difficult and takes a long period of time [7]. In addition, some AMPs can inhibit cell wall, nucleic acid, and protein synthesis at a lower concentration than the minimum inhibitory concentration (MIC) [8]. These mechanisms of action make them promising candidates for alternative antimicrobial agents.

Among AMPs, the most relevant and largest family of defense compounds is defensins, highly stable, small (45–54 amino acids) and basic cysteine-rich, cationic host defense peptides. They contribute significantly to host defense against pathogens in various organisms, including plants, invertebrates and vertebrates [9]. Plant defensins show broad biological activities including antifungal, antibacterial, antiparasitic and anticancer activities [10]. They share a common three-dimensional structure comprised of triple-stranded, antiparallel β-sheets with an α-helix in parallel, stabilized by disulfide bridges of conserved cysteine residues [11]. Despite their structural similarities, plant defensins have great diversity in amino acid sequence. This variation in the primary sequence is associated with the specificity and diverse biological activities of AMPs such as antibacterial, antifungal, anticancer, antioxidant, antiparasitic, insect growth inhibitory, protease inhibitory or HIV reverse transcriptase inhibitory activities [12]. Recently, a conserved γ-core motif (GXCX_3-9_C) composed of two antiparallel β-sheets and an interposed loop has been identified and shown to be important for their functions [13]. Two plant defensins, MsDef1 and MtDef4, from *Medicago sativa* both contain a highly conserved γ-core motif though they differ in net positive charge. When the γ-core motif of MsDef1 was replaced with that of MtDef4, it became almost as potent as MtDef4. This change also caused it to lose its mode of antifungal action, inducing hyperbranching of fungal hyphae [13]. A 17-mer SolyC peptide corresponding to the γ-core motif of tomato defensin inhibita several human pathogens including *Staphylococcus aureus*, *S*. *epidermidis*, *Listeria monocytogenes*, and *Helicobacter pylori* [14].

Using a liposome pull-down assay, the plant defensin NaD1 is found to specifically bind to phospholipids, especially phospholipid phosphatidylinositol 4,5-bisphosphate (PIP_2_) but not to sphingolipids. The crystal structure of a complex of NaD1 and PIP_2_ (NaD1:PIP_2_) revealed that the dimerization of NaD1 leads to the formation of a cationic grip which is able to accommodate two negatively charged head groups of PIP_2_. The binding site is formed by the Lys^4^ residue and KILRR motif (K^36^, I^37^, L^38^, R^39^, and R^40^ residues) [15]. The specific interaction of NaD1 with cellular phospholipid membranes, PIP_2_, is a key component of the membrane disorder [16]. This disorder can increase permeability of the microbial membrane and cause membrane disruption, resulting in cell death. Using confocal microscopy, NaD1 accumulated on the surface of *C*. *albicans* cells within 5 minutes but did not impair viability. After 20 min, NaD1 had entered the cytoplasm of cells together with propiodium iodide, thus indicating that the integrity of the plasma membrane was compromised [17].

Thailand is rich in diverse plant resources including medicinal plants. As far as we are aware, there are no studies on defensins from Thai medicinal plants. The present report describes the successful cloning and characterization of a novel defensin gene from *Brugmansia* x *candida* (*BcDef*), a plant used in traditional Thai medicine as an antispasmodic and anodyne. The 3D structure of BcDef was predicted by homology modeling in order to understand the structure-function relationship. A short peptide (BcDef1) corresponding to its γ-motif was chemically synthesized and tested for antimicrobial activity and mechanisms of action.

## Materials and methods

### Bacterial strains and culture conditions

*Escherichia coli* strain DH5α was used for all cloning experiments. Strains were cultivated in LB medium at 37°C. When necessary, the medium was supplemented with ampicillin (100 μg/ml). *Enterococcus faecalis* ATCC 29212, *Bacillus cereus* ATCC11778, *Staphylococcus aureus* ATCC 25923, *Staphylococcus epidermidis* ATCC 12228, *E*. *coli* ATCC 25922, *Pseudomonas aeruginosa* ATCC 27853, *Shigella sonnei* ATCC 11060 and *Salmonella* Typhimurium ATCC 13311 strains were grown in Tryptic soy broth (TSB, Difco, USA) at 37°C.

### Molecular cloning of defensin gene from *Brugmansia* x *candida* (*BcDef*)

*Brugmansia* x *candida* was collected at the Nawarat Botanical Garden in Pathumthani, Thailand. This study was carried out on private land with the permission from the owner of the land. No specific permissions were required for this location/activity. This study did not involve endangered or protected species. The plant was identified by botanist at the Queen Sirikit Botanic Garden Herbarium. The voucher specimen was prepared and deposited in the Queen Sirikit Botanic Garden Herbarium. Total RNAs were isolated from leaves, stems, and flowers of *B*. x *candida* by using InviTrap^®^ spin plant RNA mini-kit (STRATEC, Germany). Plant defensin cDNA was cloned by a two-step reverse transcriptase polymerase chain reaction (RT-PCR) using the degenerated primer SolaDefF1 [F: 5'-ATGGCACA(A/C)TC(T/C)AT(GTC)CGTTT(G/C)TTTGC-3'] and oligo dT. The PCR conditions were: 98°C for 30 sec, followed by 35 cycles of amplification (98°C for 10 sec, 52°C for 30 sec, 72°C for 45 sec) and final extended at 72°C for 10 min. The PCR-amplified products were purified by E.Z.N.A. Gel Extraction Kit (Omega Biotek, USA) and cloned into pTG19-T vector (Vivantis, Malaysia). The positive clones were identified through restriction digest with *Bam*HI and the sequences were determined using M13F forward primer. The nucleotide sequence of *BcDef* was submitted to GenBank under accession number MG923958.

### Sequence analysis and in silico characterization

The nucleotide sequence was compared with the GenBank database using BLASTX search. The deduced amino acid sequence of the plant defensin gene was analyzed using ORF finder (https://www.ncbi.nlm.nih.gov/orffinder/) and computer analyses of the amino acid sequence were performed with the ProtParam tool on the ExPASy server (http://www.expasy.org). In order to predict the presence of a signal peptide, SignalP 4.1 server was used (http://www.cbs.dtu.dk/services/SignalP/). Possible disulfide bridges were determined using the DISULFIND server (http://disulfind.dsi.unifi.it/). Amino acid alignment was performed using ClustalW and formatted using the Jalview program. This alignment result was then used for a Maximum-Likelihood phylogenetic analysis using MEGA7 (version 7.0) program [18] to investigate evolutionary relationships.

### Homology modeling

The structure of BhDef was constructed by a homology modeling method using SWISS-MODEL server (https://swissmodel.expasy.org). In order to predict a structural model with an accuracy equivalent to a crystallographic structure, a template with over 30% sequence identity to a target protein was used for model building [19]. Firstly, loop structures of templates were applied to the amino acid sequence of plant defensin for loop modeling. Then, side chains were computed and generated using a backbone-dependent rotamer library [20]. Finally, energy minimization was performed using WinCoot program version 0.8.6 [21] for structural model refinement and these refined models were evaluated for quality. The 3D models were validated using a PROCHECK server [22–23] (http://www.ebi.ac.uk/thornton-srv/software/PROCHECK/) by verifying the parameters of Ramachandran plot quality [24].

### Design of short synthetic peptide

Short peptide derivative of the novel plant defensin, BcDef1, was designed from the predicted important functional regions by *in silico* identification using multiple sequence and structural alignments. The peptide BcDef1 (FSGGDCRGLRRRCFCTR-NH_2_) was chemically synthesized using an Fmoc/tBu solid phase procedure provided by China peptide (Shanghai, China). The molecular mass and purity of the purified peptides (with purity more than 98%) were verified by mass spectroscopy and reversed-phase high-performance liquid chromatography (RP-HPLC), respectively. Additional physicochemical properties, including hydrophobicity and net charge at neutral pH, were determined by using the Heliquest server on-line (http://heliquest.ipmc.cnrs.fr/cgi-bin/ComputParamsV2.py) [25].

### Antimicrobial susceptibility assay

Minimum inhibitory concentrations (MICs) were determined using a modified broth microdilution assay following the Clinical and Laboratory Standards Institute (CLSI) guidelines [26]. Briefly, 50 μl (10^5^ CFU/well) of bacterial cells grown in Mueller Hinton Broth (MHB) was mixed with an equal volume of BcDef1 (0 to 251.21 μM) in 96-well plates and incubated at 37°C for 24 h. The MIC was defined as the lowest concentration of a peptide that inhibited growth of the bacteria after overnight incubation. Each experiment was performed at least three times for each species of bacteria.

### *In vitro* antioxidant activity determination

Total antioxidant status of BcDef1 was measured using a 2,2′-azinobis[3-ethylbenzthiazoline-6-sulphonic acid] (ABTS) assay as previously described [27]. Briefly, different concentrations (0–25 μM) of BcDef1 and glutathione (GSH) (Sigma-Aldrich, USA) (50 μl/well) were mixed with diluted ABTS· radical solution (200 μl/well). Absorbance was monitored at 734 nm and used for calculating the Trolox equivalent antioxidant capacity (TEAC). For the DPPH method, the peptide and GSH were transferred into 96-well microplates (100 μl/well), and mixed with 100 μM DPPH (Sigma-Aldrich, USA) (100 μl/well). The reaction was incubated at room temperature in the dark with shaking for 30 min and the absorbance at 517 nm was measured by microplate reader. The percentage of DPPH decolourization of the samples was calculated [28].

### Mammalian-cell cytotoxicity

L929 mouse fibroblast cells were grown in Roswell Park Memorial Institute (RPMI)-1640 medium supplemented with 10% (v/v) fetal bovine serum (FBS), 0.2% (v/v) sodium bicarbonate, 0.37% (wt/vol) NaHCO_3_, 100 IU/ml penicillin, and 100 μg/ml streptomycin (Biochrom AG, Germany). 1×10^4^ L929 cells/well were seeded into 96-well plates and incubated at 37°C in an air atmosphere containing 5% CO_2_ for 24 h. Then cells were treated with different concentrations of BcDef1 (0–100.49 μM) for 24 h. MTT solution (5 mg/ml) was added to each well and incubated for 3.5 h at 37°C. Then, supernatant was discarded and 150 μl of DMSO was added to each well and mixed gently. The absorbance at 590 nm of the reaction solution was measured by microplate reader. The Probit analysis was applied to determine the IC_50_ value.

### Transmission electron microscopy (TEM)

*S*. *epidermidis* at exponential growth phase were treated with BcDef1 at the 0.5×MIC, and incubated at room temperature (25°C) for 2 h. Untreated cells were used as controls. Both treated and untreated cells were collected by centrifugation at 10,000×g for 20 min, washed with Ringer's solution and fixed with 2.5% glutaraldehyde in 0.1 M sodium phosphate buffer pH 7.2 at 4°C for 12 h. Then, samples were processed for TEM (HT7700, Hitachi, Japan) observation.

### Flow cytometric analysis

*S*. *epidermidis* at exponential growth phase were treated with BcDef1 at the MIC, and incubated at room temperature (25°C) for 30 or 60 min. Untreated cells and thermal lysis cells (70°C for 30 min) were used as negative and positive controls, respectively. After that, cells were collected and resuspended in 1 ml of Ringer's solution. Propidium iodide (PI, 10 μg/ml) and bis-(1,3-dibutylbarbituric acid) trimethine oxonol (BOX, 0.5 μM) were added and the fluorescent intensity of each sample was measured by BD FACSCalibur™ flow cytometry (BD Biosciences, USA). The data were analyzed with FlowJo version 10.1 (LLC, USA).

### Statistical analysis

All the experiments were performed in triplicate and the data were expressed as the mean ± SD. The statistical analyses were performed by one-way ANOVA and Tukey's test at p < 0.05 level using SPSS program version 14.0.

## Results

### Sequence features and phylogenetic tree analysis of defensin gene from *B*. x *candida*

**Fig 1:**
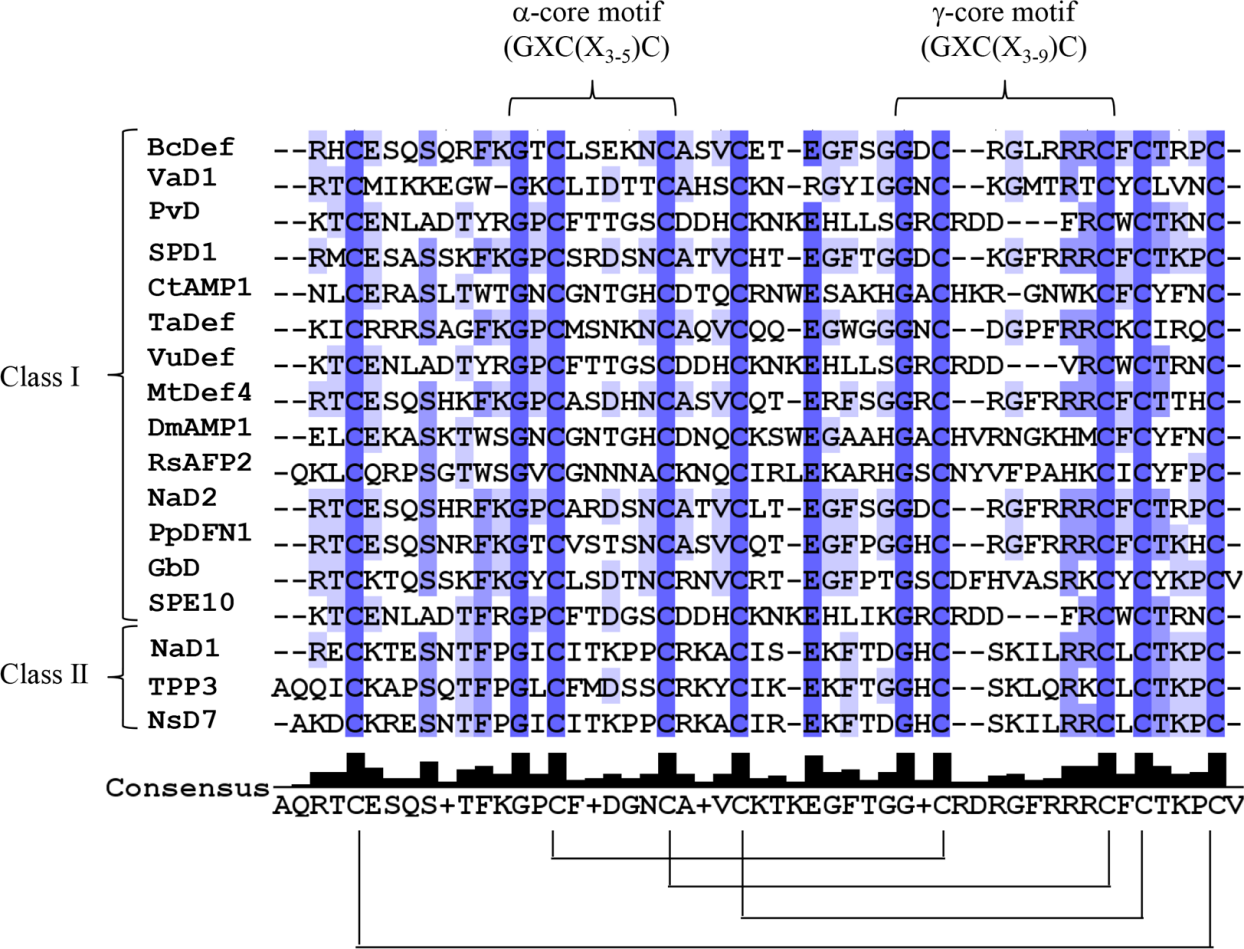
Multiple alignment of plant defensins. Shading indicates sequence identity from low (light blue) to high (dark blue). The brackets indicate the conserved α-core and γ-core motifs consensus sequence, respectively. Disulphide bonds are depicted by connecting lines.

Using RT-PCR, the full-length cDNA sequence encoding a novel defensin gene was obtained from stem of *B*. x *candida* and designated as *BcDef*. The cloned *BcDef* cDNA was 237 bp in length encoding a 78-amino acid peptide, with a putative 31-amino acid signal peptide at the N-terminal followed by the mature peptide sequence. It belongs to the class I plant defensins, characterized by an endoplasmic reticulum (ER) signal sequence and lack of the C-terminal propeptide characteristic of class II defensins. The calculated molecular mass of mature BcDef without signal peptide was 5.29 kDa with a theoretical pI of 8.76, indicating a net cationic charge which is generally characteristic of AMPs. Further sequence alignment revealed that BcDef shared high sequence identity (78–85% homology) with Solanaceae defensins and the presence of α-core and γ-core motifs with consensus sequences GXC(X_3-5_)C and GXC(X_3-9_)C, respectively (Fig 1). The net charge of the γ-core motifs was higher than that of the α-core; the γ-core motif of BcDef has total net charge of +3.

**Fig 2:**
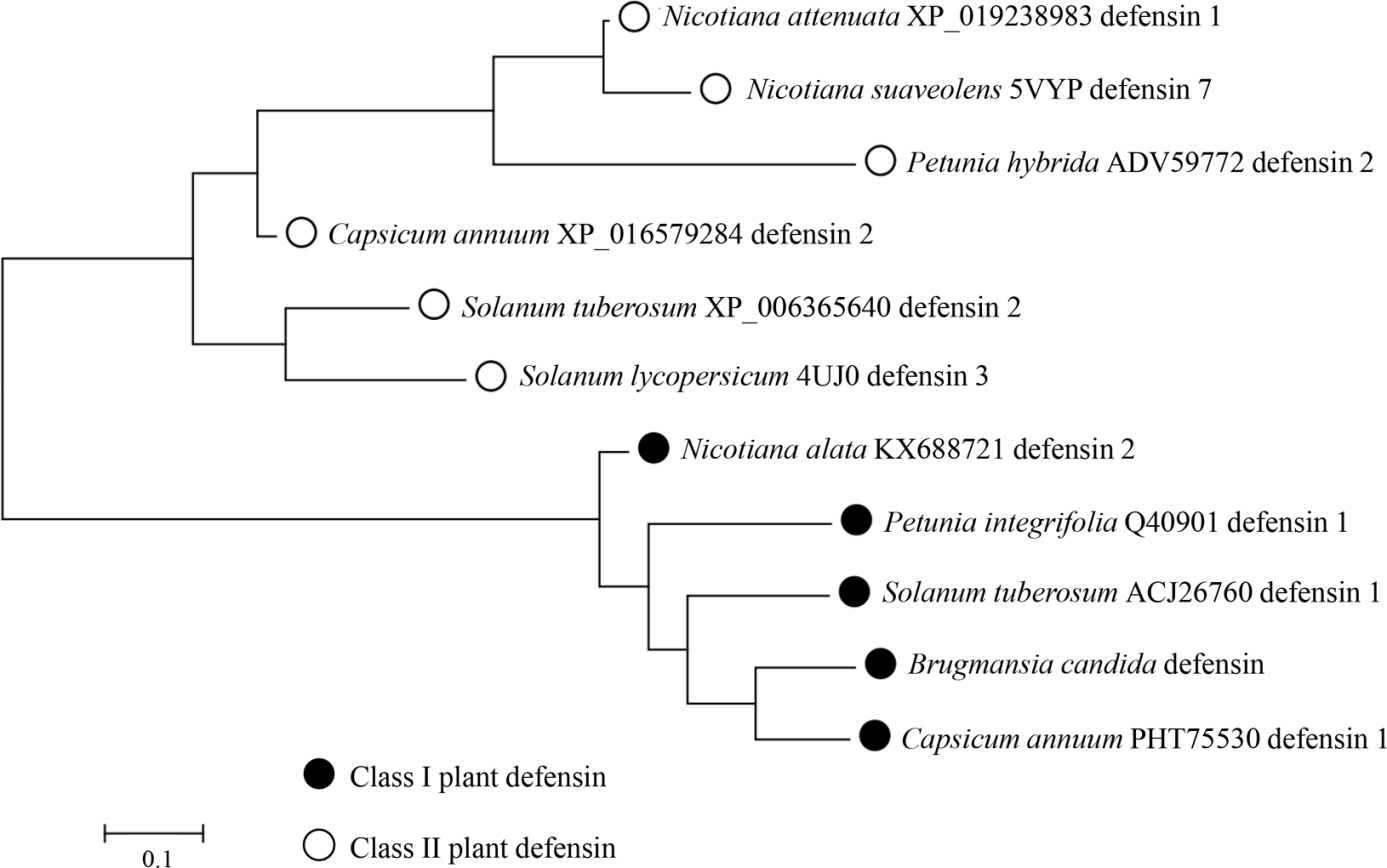
Maximum Likelihood phylogenetic tree of BcDef and defensins of the Solanaceae plant family.

The amino acid sequences of plant defensins are highly diverse. Only the eight cysteine (C), two glycine (G) and one aromatic residues (phenylalanine; F, tyrosine; Y and tryptophan; W) were conserved. These eight conserved cysteines are signature residues of defensins and involved in disulfide bonds essential for structural folding. As predicted by the DISULFIND server, BcDef contained a tetradisulfide array similar to other plant defensins (Fig 1). A Maximum Likelihood phylogenetic analysis (Fig 2) of BcDef and defensins of the Slanaceae plant family revealed that phylogenetic tree was divided into two main clusters, class I and class II defensins. Class I defensins were further divided into two sub-clusters, one sub-cluster included *Nicotana attenuata* defensin 1, *N*. *suaveolens* defensin 7, *Petunia hybrida* defensin 2 and *Capsicum annuum* defensin 2, and another sub-cluster contained *Solanum tuberosum* defensin 2 and *S*. *lycopersicum* defensin. BcDef was placed in the sub-cluster of class I defensins together with *N*. *alata* defensin 2, *Petunia integrifolia* defensin 1, *S*. *tuberosum* defensin 1 and *C*. *annuum* defensin 1. BcDef was closely related to Ca1 (*C*. *annuum* defensin 1). The results suggested that BcDef belongs to class I plant defensins.

### The structure-function relationships analysis of BcDef peptide

**Fig 3:**
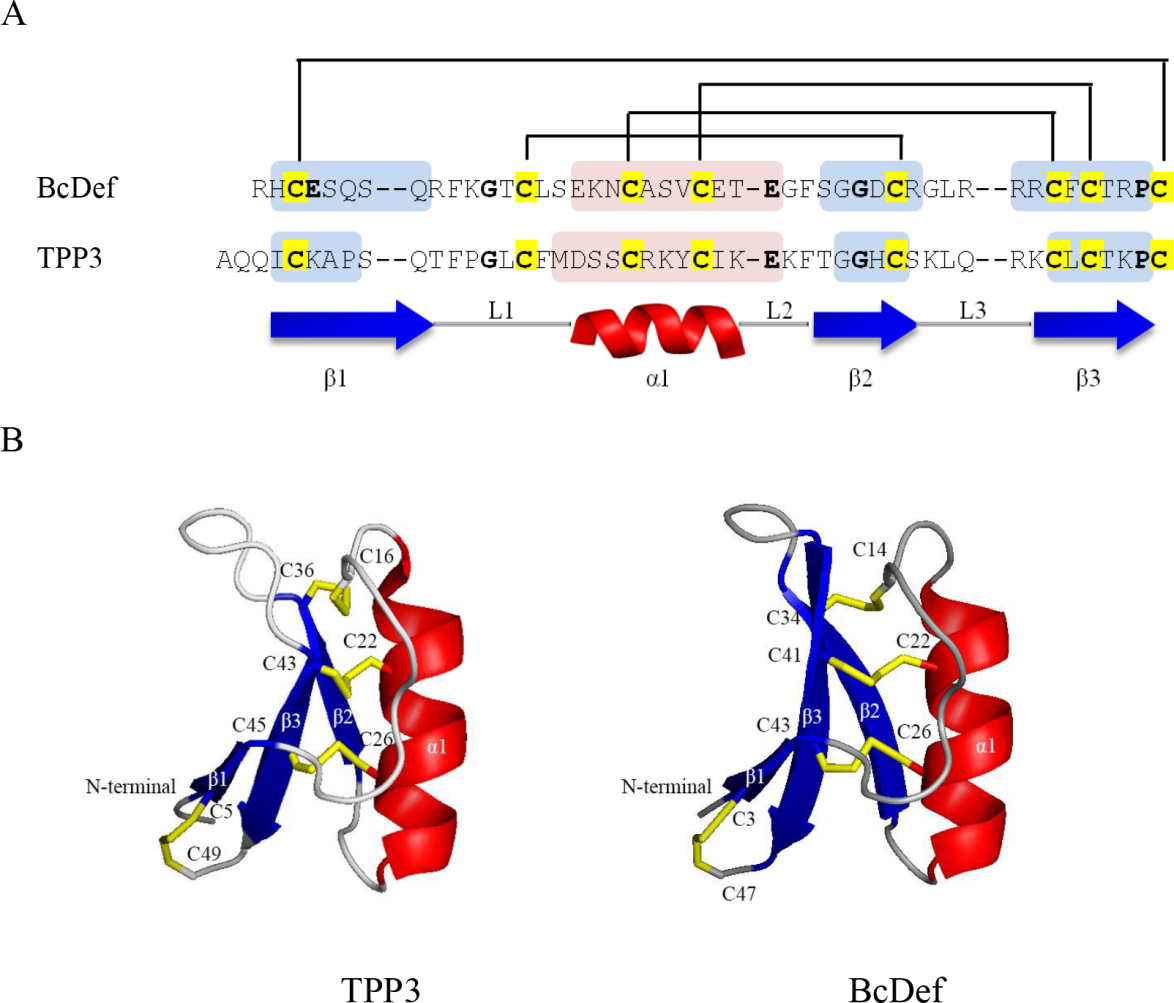
Amino acid sequences and the 3D structural models of BcDef and TPP3. (A) Sequence alignment of target sequence, BcDef, with template sequence, TPP3 (PDB: 4UJ0). Disulphide bonds are depicted by connecting lines. The conserved residues are shown in bold and the eight conserved cysteine residues are highlighted in yellow. The β-strands and α-helices are indicated underneath and within the sequences by blue and red shading, respectively. (B) The structures of TPP3 monomer and BcDef. All peptides share a conserved triple stranded β-sheet (blue) and one α-helix (red). Their secondary structures are held together by four disulphide bridges (yellow sticks).

The homology models of BcDef were generated to predict three-dimensional structures and can further contribute to understanding the relationships between their structures and functions. By using ClustalW, BcDef shared the highest sequence identity (42.55%) with TPP3 (PDB: 4UJ0). The BcDef structural model was then built based on a TPP3 template (Fig 3); it shared a conserved triple-stranded β-sheet (β1-β3) and one α-helix (α1) which were connected by loops L1, L2, and L3. Its secondary structure was held together by four disulphide bridges. Three of them formed conserved intramolecular disulphide bridges which have the general fold of the cysteine-stabilized αβ (CSαβ) motif [29]. The other disulphide bond brings together the N- and C-terminal regions of the molecule, forming a pseudocyclic structure. The major differences are loop length between β1 and α1, and between β2 and β3. To identify conserved functional motifs, the consensus sequence of the γ-core motif (GXCX_3-9_C) was used. The electrostatic surface of each model was calculated by PyMOL program (S1 Fig). The γ-core motif of BcDef is located in loop 3 (GDCRGLRRRC) and has strongly positive surface potentials and might influence their function.

To predict the structure-function relationships, the structural model of BcDef was superimposed on other known functional plant defensins MtDef4 (PDB: 2LR3), NaD1 (PDB: 4AB0) and RsAFP2 (PDB: 2N2R)] using PyMOL program. These plant defensins have been reported to exhibit antifungal activity mediated by specific protein-lipid binding interactions. Despite their differences in specific lipid binding sites, these antifungal plant defensins share highly conserved tertiary structures (S2 Fig). The BcDef structural model was superimposable on MtDef4, NaD1 and RsAFP2. Notably, there are differences in only the loop regions which have been reported as a functionally important region of plant defensins [13–14, 30]. The loop 3 region is important for lipid binding which is a crucial step in the mechanism of action of most plant defensins [15]. This result suggests that the specific lipid binding properties of BcDef probably differ from those of MtDef4, NaD1, and RsAFP2. Additionally, the other important factor affecting the protein-lipid interaction is surface charge. As shown in S1 Fig, the positive charges spread out over the surface of the peptide, especially the surface around loop 3. These positively charged sites are reported to play an important role in the antimicrobial activity of plant defensins [15, 31]. The antimicrobial activities NaD1 and TPP3 plant defensins are mediated by PIP_2_ binding. They interact with negatively charged phospholipid head groups via their positively charged surfaces.

### Design of BcDef1 and its antimicrobial activity

In order to evaluate the importance of the loop 3 region for the antimicrobial activity, the peptide derivative, BcDef1, was designed. In addition to the importance of structural unit, the peptide was also designed based on primary amino acid sequences that exhibited antimicrobial activity. BcDef peptide was aligned with those of other plant defensins with regions known to be important for their antimicrobial activity such as the 16-mer (GMA4-C), 19-mer (MBG01) and 17-mer SolyDef peptides (S3 Fig). Moreover, the amino acid residues involved in lipid membrane binding of the antifungal defensins NaD1, TPP3 and NsD7 have been identified in a similar region to that of GMA4-C, MBG01 and SolyC. Based on these alignments, the 17-mer BcDef1 (FSGGDCRGLRRRCFCTR-NH_2_) was designed from BcDef (S4 Fig). Its antibacterial activity was determined and compared with that of melittin (used as a reference peptide in the present study, Table 1). BcDef1 showed antibacterial activity against both Gram-negative and Gram-positive bacteria, including *E*. *coli*, *V*. *cholerae*, *S*. *sonnei*, *S*. *typhimurium*, *E*. *faecalis*, *B*. *cereus* and *S*. *epidermidis*. BcDef1 showed the highest activity against *S*. *epidermidis* with an MIC value of 15.70 μM. In comparison, the MIC of the synthetic peptide SolyC, designed from the γ-core motif of tomato defensin against *S*. *epidermidis*, was 40 μg/ml [14].

**Table 1:** Minimum inhibitory concentration (MIC) of synthetic BcDef1.

Bacterial strain		MIC (μM)	
		BcDef1	Melittin
Gram-negative	*E*. *coli* ATCC 25922	>251.21	2.20
	*E*. *coli* O157	229.09	4.41
	*P*. *aeruginosa* ATCC 27853	>251.21	8.81
	*V*. *cholerae* O1 Inaba	125.61	2.20
	*S*. *sonnei* ATCC 11060	125.61	2.20
	*S*. *typhimurium* ATCC 13311	31.40	2.20
Gram-positive	*E*. *faecalis* ATCC 29212	251.21	2.20
	*B*. *cereus* ATCC 11778	251.21	2.20
	*S*. *aureus* ATCC 25923	>251.21	2.20
	MRSA ATCC 43300	>251.21	2.20
	*S*. *epidermidis* ATCC 12228	15.70	1.10

### *In vitro* antioxidant activity determination of the BcDef1 peptide

The scavenging activities of BcDef1 and GSH act in a concentration-dependent manner. The concentration required to scavenge DPPH radical by 50% (IC_50_) was determined for each (BcDef1 and GSH) from the linear equation (y = ax + b). The concentration of BcDef1 required to scavenge DPPH radical by 50% (IC_50_) was 5.84 μM which was 8.41 times higher than that of GSH (49.11 μM). BcDef1 had its highest scavenging activity (82.34 ± 0.85%) at the concentration of 25 μM, even higher than the highest GSH activity (40.54 ± 0.86) (Table 2). Using the TEAC method, BcDef1 and GSH exhibited dose-dependent antioxidant activity. At the concentrations of 5, 10, 15, and 20 μM, BcDef1 had significantly higher TEAC values than did GSH (p≤0.05) (Table 2). These results indicated that BcDef1 exhibits antioxidant activity and its activity is greater than that of GSH.

**Table 2:** Antioxidant activity of BcDef1 and glutathione.

Concentration (μM)	DPPH radical scavenging activity (%)		TEAC (μM TE)	
	BcDef1	Glutathione	BcDef1	Glutathione
0	0.00 ± 0.00	0.00 ± 0.00	0.00 ± 0.00	0.00 ± 0.00
5	42.73 ± 1.79*	14.03 ± 0.62*	17.53 ± 0.69*	10.16 ± 0.61*
10	67.31 ± 2.11*	23.36 ± 0.90*	31.99 ± 0.32*	20.25 ± 0.27*
15	77.44 ± 0.88*	28.96 ± 0.91*	39.11 ± 0.86*	27.98 ± 1.33*
20	81.13 ± 1.12	35.60 ± 0.92*	39.52 ± 0.86*	35.34 ± 0.79*
25	82.34 ± 0.85	40.54 ± 0.86	39.85 ± 1.02	38.52 ± 1.35

The values are expressed as the mean ± SD of three experiments.

* indicate significant differences

### *In vitro* cytotoxicity determination of the BcDef1 peptide

At the concentrations of 0.5, 5.02, 50.24, and 100.49 μM, the viabilities of BcDef1-treated L929 mouse fibroblast cells were significantly lower than that of control cells (p≤0.05). The IC_50_ value of BcDef1 was found to be 140.76 μM as determined by probit analysis [32]. It was 8.97 times higher than MIC value of BcDef1 against *S*. *epidermidis* (15.70 μM).

### Mechanism of action determination of the BcDef1 peptide

**Fig 4:**
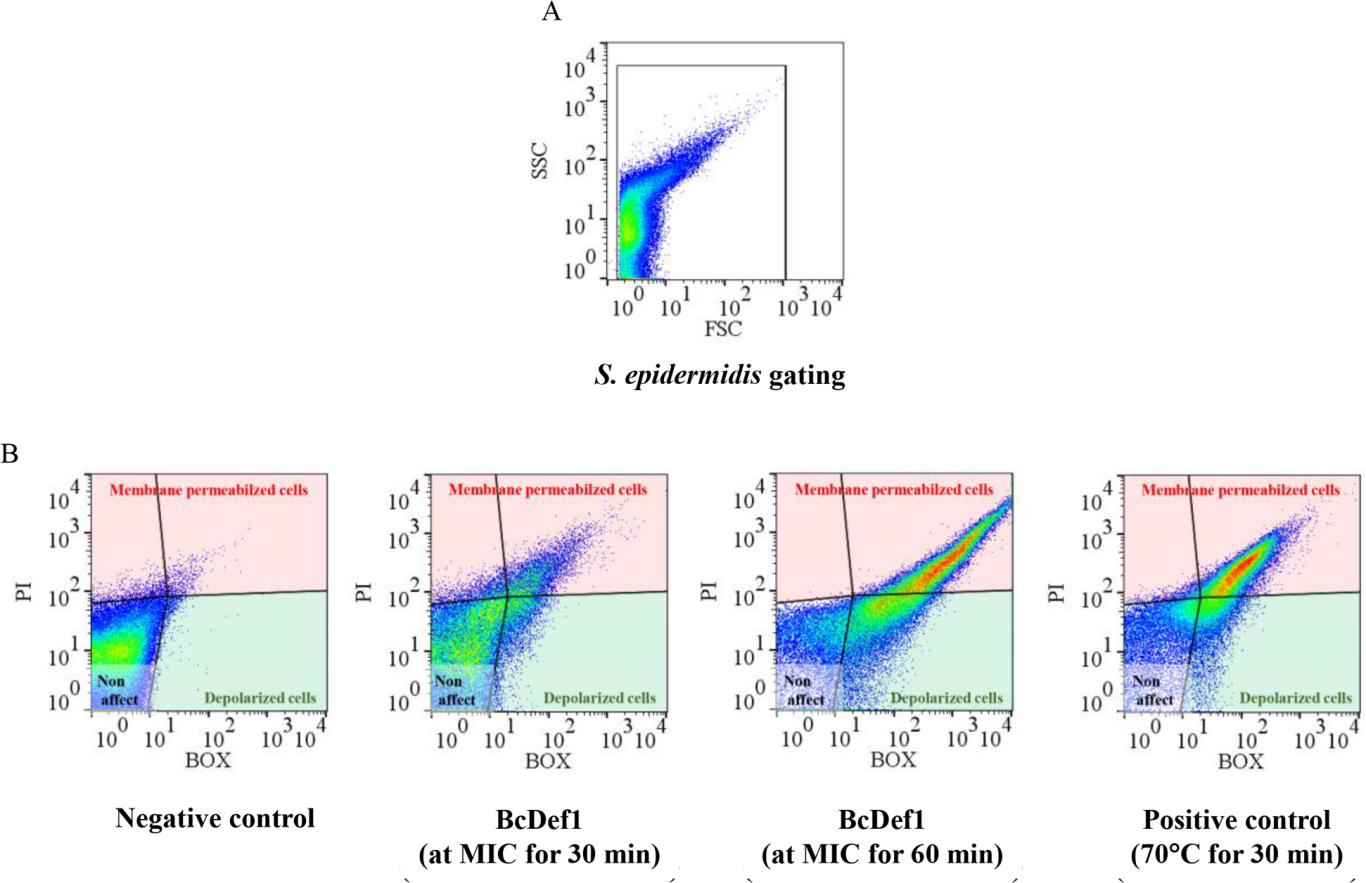
Flow cytometry analysis of *S*. *epidermidis* treated with BcDef1. (A) The *S*. *epidermidis* cell population was selected on a dot plot of FSC versus SSC. (B) The effect of BcDef1 and thermal lysis (positive control) on the membrane permeability (PI) and membrane potential (BOX) of *S*. *epidermidis* were identified and gated for PI (red gate) and BOX (green gate).

The mechanism of antibacterial action of BcDef1 was investigated at the cell membrane level using flow cytometry and transmission electron microscopy. The fluorescent stains propidium iodide (PI) and bis-(1,3-dibutylbarbituric acid) trimethine oxonol (BOX) were used to evaluate membrane permeability and membrane potential changes (depolarization) of the cell, respectively [33]. *S*. *epidermidis* cells were treated with the MIC of BcDef1 (15.70 μM) for 30 or 60 min at room temperature (25°C). Untreated cells and thermally lysed cells (70°C for 30 min), were used as negative and positive controls, respectively. Each sample was stained with both BOX and PI, and fluorescent intensity of each sample was measured by FASC-flow cytometry. Fluorescence was presented in a dot pot of green fluorescence (BOX) versus red fluorescence (PI). Three regions, including membrane permeabilized, depolarized and non-affected cells, were defined by single-stained populations, PI and BOX, and the non-stained population, respectively. As shown in Fig 4, BcDef1 induced high levels of membrane depolarization and permeabilization. The cells treated with BcDef1 for 30 min had a greater percentage of depolarized (4.70 ± 0.97%) and permeabilized cells (17.21 ± 2.75%) than did untreated cells (2.08 ± 1.57 and 2.79 ± 0.61%, respectively). When the cells were incubated with BcDef1 for a longer time (60 min), the percentage of depolarized and permeabilized cells were further increased (10.70 ± 1.10 and 20.80 ± 2.98, respectively). These results suggested that BcDef1 altered both membrane potential and permeability of *S*. *epidermidis* cells in a time-dependent manner.

**Fig 5:**
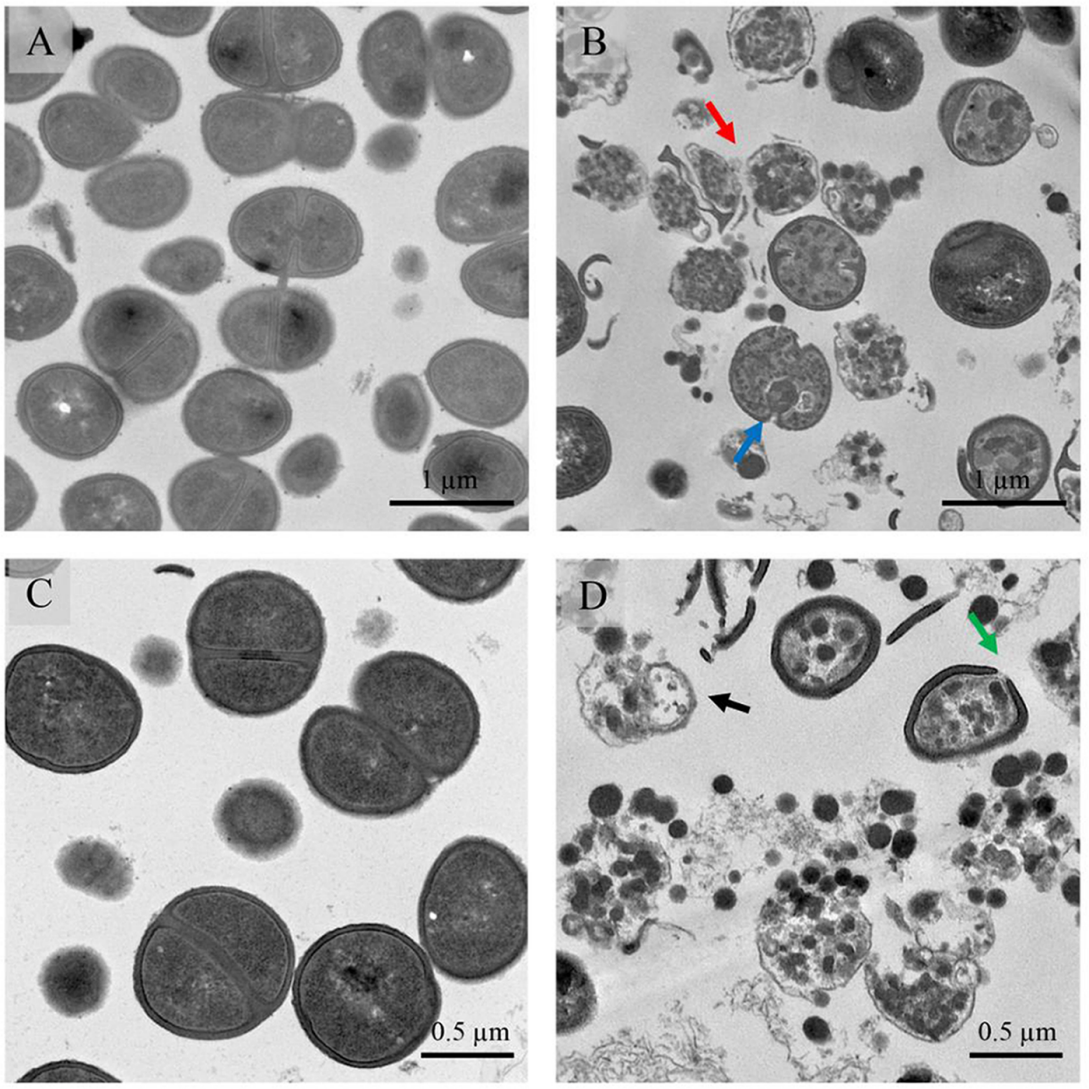
TEM micrographs of *S*. *epidermidis* after treatment with BcDef1. (A and C) Untreated cells possessed intact cell walls and cell membranes. After 2 h of BcDef1 treatment, (B and D), BcDef1 induced cell lysis (black arrow) and ultrastructural damage in *S*. *epidermidis* cells, including some mesosome-like structure formation (blue arrow), and ruptures of cell walls (green arrow) and cell membranes (red arrow).

**Fig 6:**
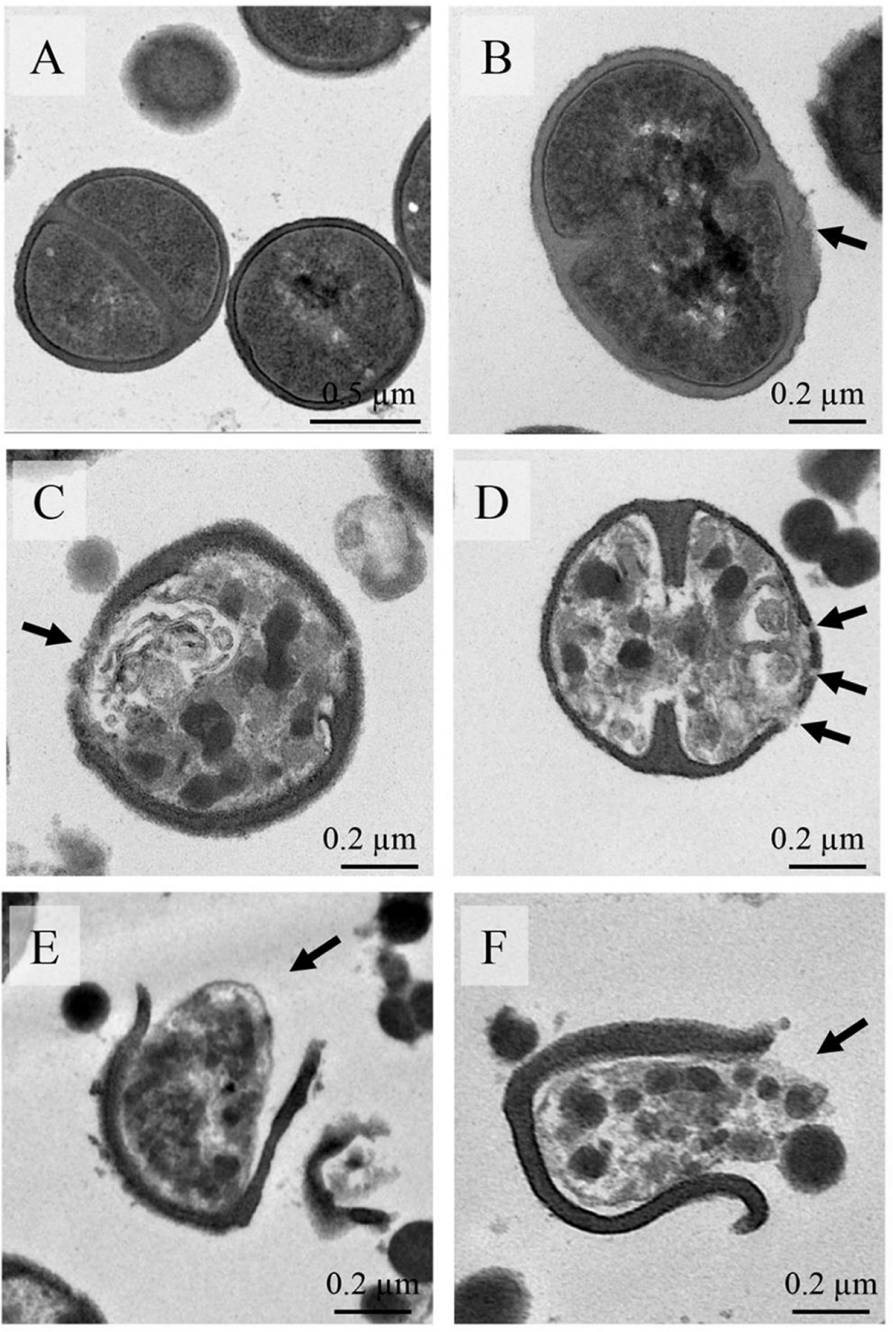
Higher magnification pictures show the structural observations of *S*. *epidermidis* treated with BcDef1. (A) Untreated cells possessed intact cell wall and cell membrane. After 2 h of BcDef1 treatment, several structural alterations were observed, including changes in cell wall thickness (B and C), pore formation in cell wall and cell membrane (D), cell wall and cell membrane disruption (E and F), and release of cellular content (F).

Transmission electron microscopy (TEM) was used to observe the effects of BcDef1 on the cell morphology of *S*. *epidermidis*. Untreated *S*. *epidermidis* cells showed a normal cell shape and possessed intact cell wall and cell membrane without any damage of the inner and outer membranes (Figs 5A, 5C and 6A). In contrast, *S*. *epidermidis* cells treated with 0.5×MIC of BcDef1 for 2 h showed damage to cell structures and dramatic change to cell wall and cell membrane structures. BcDef1 induced several structural alterations, including the formation of membrane blebs (Fig 5B), numerous double-layered spherical mesosome-like structures (Fig 5B), changes in cell wall thickness (Fig 6B and 6C), pore formations in cell wall and cell membrane (Fig 6D), cell wall and cell membrane disruption (Fig 6E and 6F), and leakage of cellular contents (Fig 6F). After 2 h of incubation with BcDef1, thickening of the surface of cytoplasmic membrane and membrane blebs were discernible on the *S*. *epidermidis* cells. BcDef1-treated cells also showed a retraction of the cytoplasm with obvious clear zones; the integrity of the cell membranes were disrupted with visible pores together with leakage of entire cytoplasmic contents.

## Discussion

In this study, a novel plant defensin gene was obtained from stem material of *B*. x *candida* (*BcDef*) using a degenerate primer (SolaDefF1) designed from the conserved amino acid sequences of Solanaceae defensins. As far as we are aware, this is the first report of a defensin from medicinal plants which have long been used traditionally for treating various diseases. The sequence variation in plant defensins is thought to be responsible for the specificity and diversity of their biological activities. The radish antifungal defensins, RsAFP1 and RsAFP2, showed only two amino acid differences in their sequences (Gln^5^ is Glu and Arg^27^ is Asn). However, the antifungal activity of RsAFP2 is 2–30 fold more potent against several fungi than RsAFP1 [34–35]. With a highly conserved three-dimensional structure, plant defensins are composed of three antiparallel β-strands, an α-helix, and three loops stabilized by disulphide bonds in a β1-α-β2-β3 configuration [11, 13, 29, 31].

The differences in the amino acid sequence and charge distribution of (solvent-exposed) loops in the structures have been suggested to be important for their antimicrobial activity [13, 36–37]. Many reports note that amino acid residues which are essential for functional activity, especially antimicrobial activity, are located mostly in the conserved γ-core motif [13, 36, 38] within the β2-β3 loop. In 2011, *Sagaram et al*. described the importance of the γ-core motif for antimicrobial activity in two *Medicago* defensins (MsDef1 and MtDef4) [13]. Both defensins contain a highly conserved γ-core motif which differs in net positive charge. MtDef4 has a significantly higher net positive charge (+6) and is more potent against *Fusarium graminearum* when compared to MsDef1 that has a net positive charge of +3.

The structure of BcDef from homology modeling was similar to other plant defensins. The γ-core motif, GDCRGLRRRC, was located in β2 and β3 strands connected by the positively charged loop 3. Interestingly, this peptide consists of an RGLRRR motif similar to the γ-core motif of MtDef4 [37]. The 17-mer BcDef1 designed from loop 3 of BcDef showed antibacterial activity against both Gram-negative and Gram-positive bacteria including MRSA. Its greatest activity was against *S*. *epidermidis* with an MIC of 15.70 μM.

In 2014, Poon *et al*. showed that NaD1 specifically binds to phospholipids, especially phospholipid phosphatidylinositol 4,5-bisphosphate (PIP_2_), and did not bind to sphingolipids. The dimerization of NaD1 leads to the formation of a cationic grip. This positively charged pocket is able to accommodate two negatively charged head groups of PIP_2_. The binding site is formed by the K^4^ residue and the KILRR motif [15]. This PIP_2_ binding site was not found in BcDef. This result suggests that the specific lipid-binding properties of BcDef probably differ from that of NaD1. However, BcDef contains positively-charged amino acid in the loop 3 region that could probably bind to other negatively charged lipids such as phosphatidic acid, phosphatidylglycerol, and teichoic acid. This could indicate that the novel plant defensin, BcDef, has similar potent antimicrobial activity.

Most antimicrobial peptides are supposed to kill bacteria via membrane damage [39]. The results showed that BcDef1 affected membrane potential and permeability, and caused cell membrane disruption. For the first time, chemically synthesized peptides containing the γ-core motif of this plant defensin were shown to inhibit bacterial growth via membrane damage. This was probably due to a strong electrostatic interaction between the cationic residues of BcDef1 and the anionic head groups of microbial membrane lipids. Plant defensins can specifically interact with negatively charged microbial membrane compounds such as fungal sphingolipids and phospholipids [40]. Upon the interaction, plant defensins can introduce membrane disorder and increase the permeability of the microbial membrane and cause cell disruption, resulting in cell death [41].

In addition to antimicrobial properties, some plant defensins have been reported to have antioxidant properties. The chemically synthetic peptide, CFCTKPC, of sweet potato (*Ipomoea batatas*) defensin shows such a capacity in DPPH radical scavenging and TEAC assays [28]. It is suggested that the presence of three cysteines in the peptide are responsible for the free radical scavenging [42]. BcDef1 exhibited antioxidant activity and its activity was stronger than that of reduced glutathione which is a well-known antioxidant [43]. When compared to the synthetic peptide of sweet potato defensin, CFCTKPC, BcDef1 exhibited greater antioxidant activity (IC_50_ is 5.84 μM) than that of the sweet potato defensin (IC_50_ is 11.30 μM) [28]. The cytotoxicity of BcDef1 against mammalian cells was found to be 140.76 μM which is 8.97 times higher than MIC value of BcDef1 against *S*. *epidermidis*. BcDef1 is less cytotoxic against mammalian cells than both the tomato defensin (TPP3) and NaD1. At a concentration of 10 μM, TPP3 and NaD1 induced 69.6% and 62.3% cell death of the human lymphoblast cell line U937, respectively [31].

In conclusion, a new plant defensin, *BcDef*, was identified, cloned and characterized. The synthetic peptide designed from the γ-core motif, BcDef1, exhibited antibacterial activity via membrane damage and had antioxidant activity without cytotoxicity against normal cells. These findings provide the opportunity for developing the plant defensin as a new effective antimicrobial agent and antioxidant supplement.

## Supporting information

S1 Fig Comparison of electrostatic properties of BcDef and other plant defensins. The peptide surfaces are highlighted by charge (red is negative, blue is positive and white is hydrophobic). Each peptide is presented by four electrostatic potential surface plots, representing rotation of 90° around the vertical (Z) axis. Surface representations of all peptides are shown in the same orientation as the top panel.
(TIF)

S2 Fig Comparison of the structural model of BcDef with other plant defensins. The structure of antifungal plant defensins, including MtDef4 in magenta (PDB: 2LR3), NaD1 in yellow (PDB: 4AB0) and RsAFP2 in green (PDB: 2N2R), were superimposed on the BcDef model (grey). These structure alignments show that they share highly conserved tertiary structures, although the loop regions differ.
(TIF)

S3 Fig The multiple amino acid sequence alignments of BcDef with other plant defensins. The conserved residues are shown in bold. The sequences of synthetic peptides derived from MtDef4, RsAFP2, and SolyDef are highlighted according to their activities, including antifungal (yellow) and antibacterial (grey) activities. The residues involved in lipid membrane binding of NaD1, TPP3 and NsD7 are framed in red. The region comprising BcDef1 was highlighted in red.
(TIF)

S4 Fig Representation of BcDef imposed on its own structural model. BcDef1 peptide is highlighted in red.
(TIF)
