# Supplementary figures and images for: Structural and biological features of a novel plant defensin from Brugmansia x candida
Source: PLoS One. 2018 Aug 2;13(8):e0201668. doi: 10.1371/journal.pone.0201668 (PMC6072023; doi:10.1371/journal.pone.0201668)

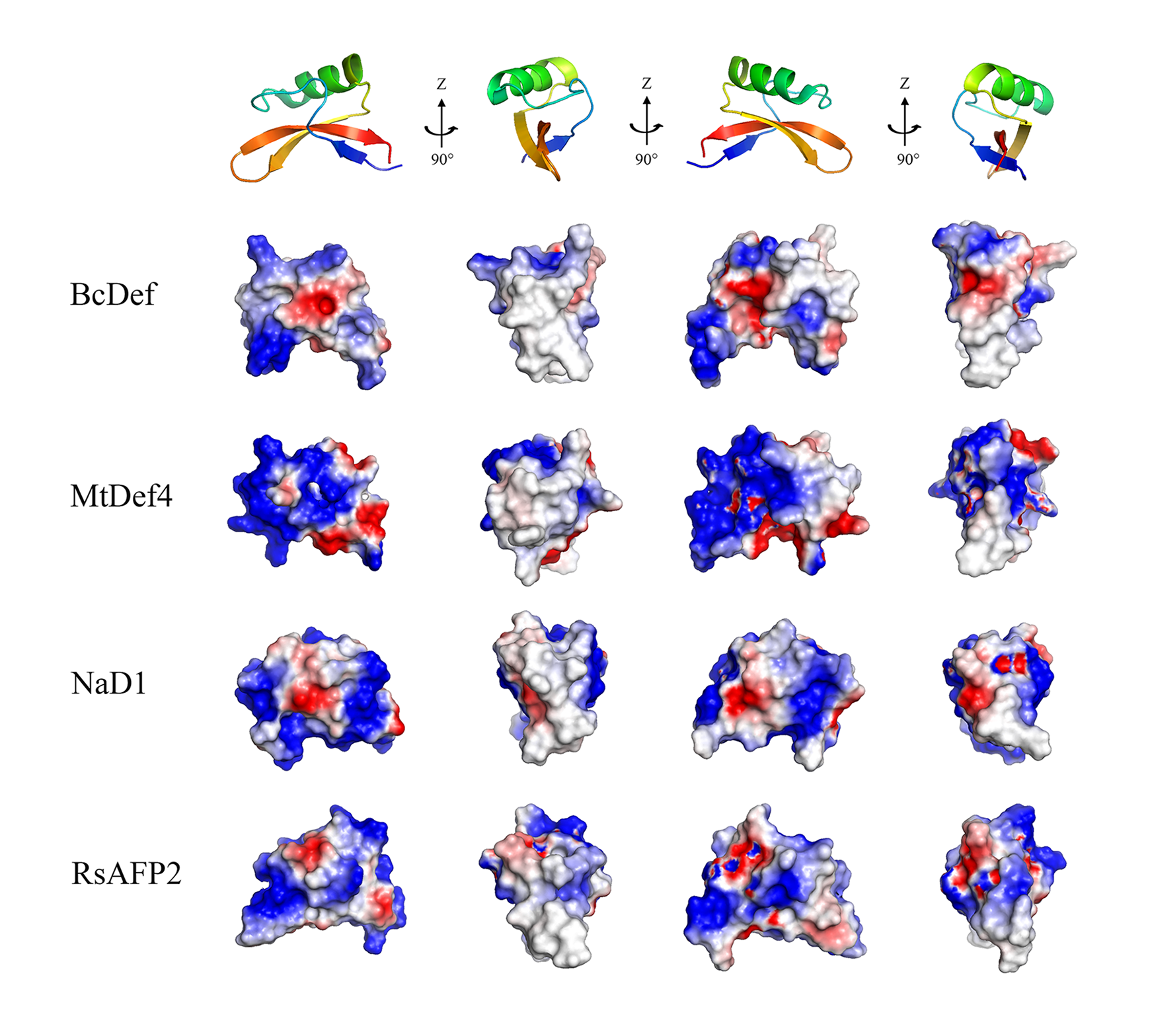

Supplement: S1 Fig — The peptide surfaces are highlighted by charge (red is negative, blue is positive and white is hydrophobic). Each peptide is presented by four electrostatic potential surface plots, representing rotation of 90° around the vertical (Z) axis. Surface representations of all peptides are shown in the same orientation as the top panel. (TIF) [file pone.0201668.s001.tif]

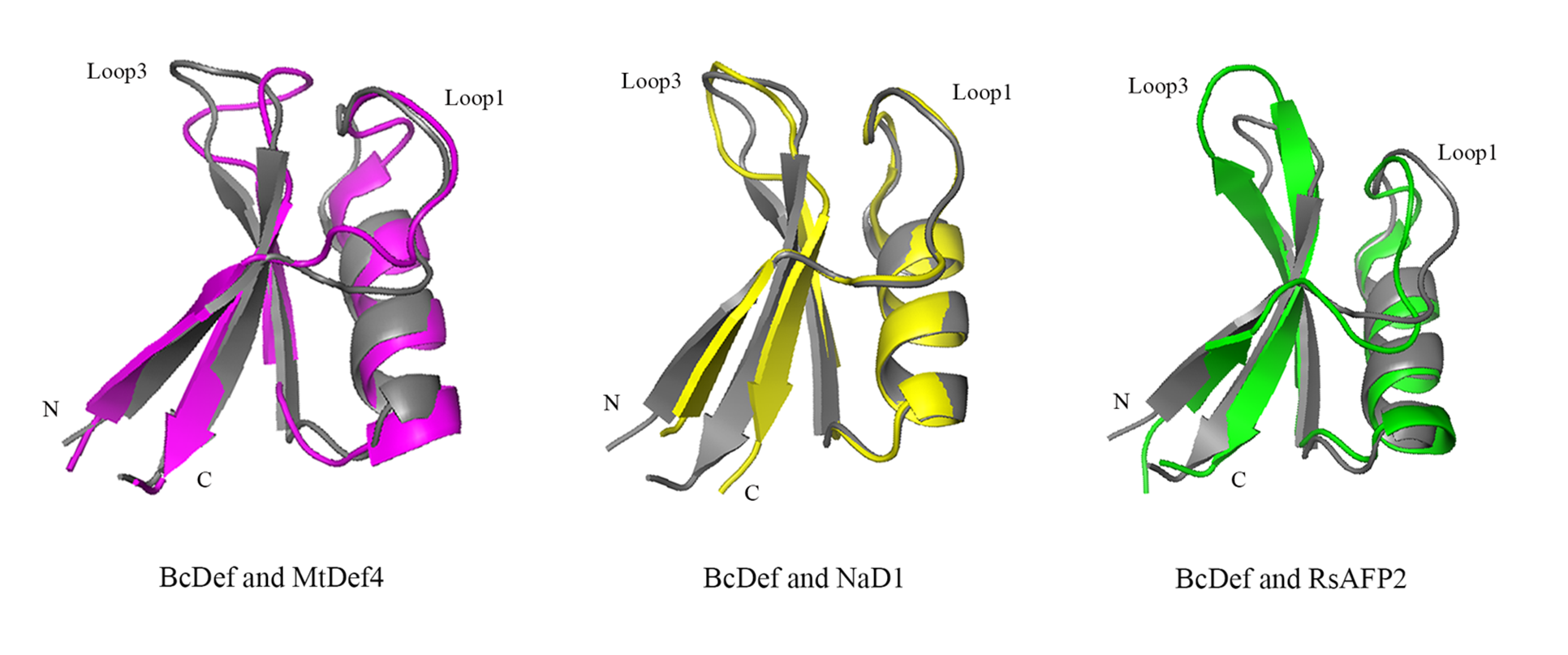

Supplement: S2 Fig — The structure of antifungal plant defensins, including MtDef4 in magenta (PDB: 2LR3), NaD1 in yellow (PDB: 4AB0) and RsAFP2 in green (PDB: 2N2R), were superimposed on the BcDef model (grey). These structure alignments show that they share highly conserved tertiary structures, although the loop regions differ. (TIF) [file pone.0201668.s002.tif]

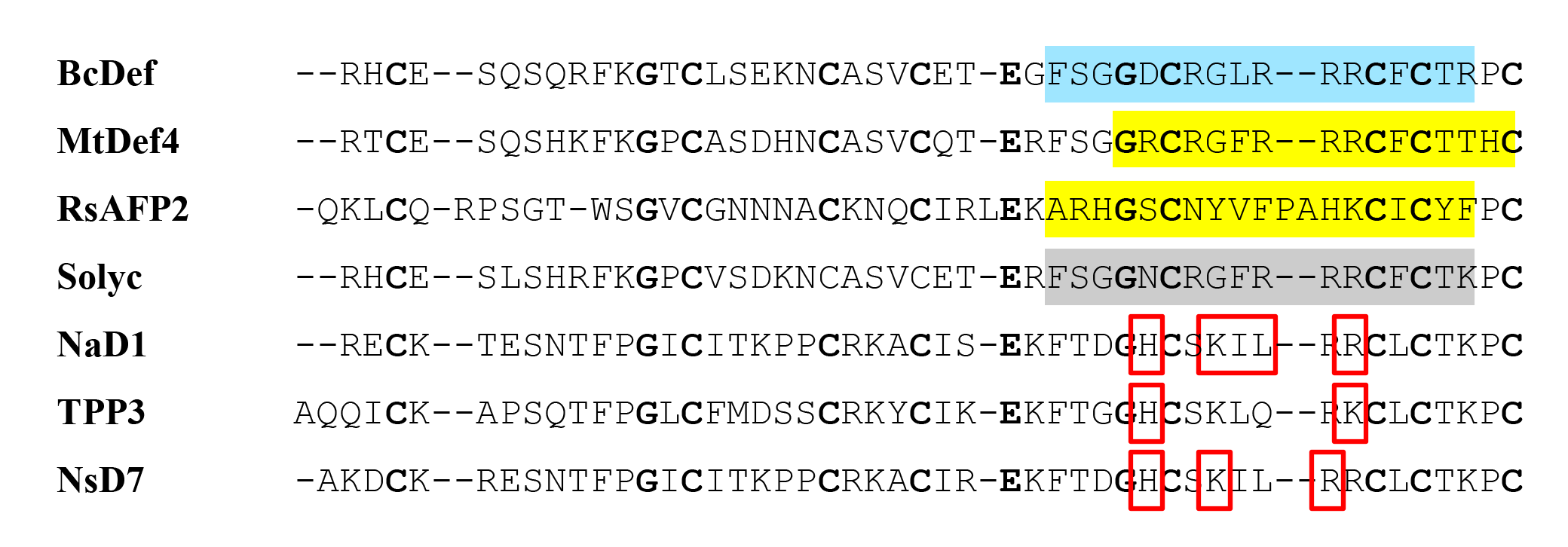

Supplement: S3 Fig — The conserved residues are shown in bold. The sequences of synthetic peptides derived from MtDef4, RsAFP2, and SolyDef are highlighted according to their activities, including antifungal (yellow) and antibacterial (grey) activities. The residues involved in lipid membrane binding of NaD1, TPP3 and NsD7 are framed in red. The region comprising BcDef1 was highlighted in red. (TIF) [file pone.0201668.s003.tif]

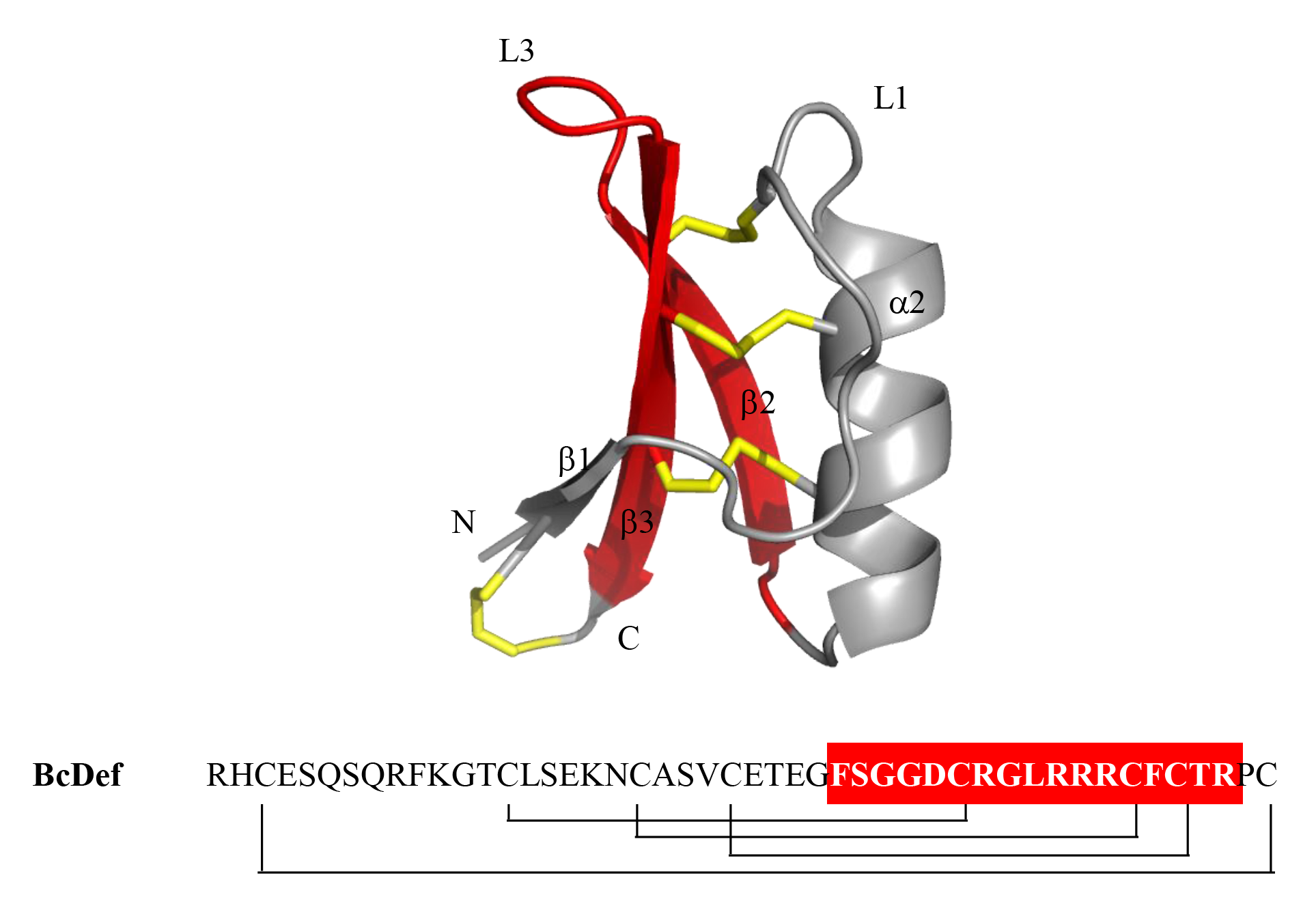

Supplement: S4 Fig — BcDef1 peptide is highlighted in red. (TIF) [file pone.0201668.s004.tif]
